# A prospective, real-world, multinational study of febrile neutropenia (FN) occurrence in oncology patients receiving chemotherapy with intermediate risk of FN: a MASCC Neutropenia, Infection, and Myelosuppression Study Group initiative

**DOI:** 10.1007/s00520-023-08071-0

**Published:** 2023-10-13

**Authors:** Bernardo Leon Rapoport, Marcial Garcia-Morillo, Carme Font, Zarka Samoon, Adnan Abdul Jabbar, Hampig Raphael Kourie, Aline Kayumba, Francis Esposito, Razvan Andrei Popescu, Jesus García-Gómez, Liezl Heyman, Teresa Smit, Andriy Krendyukov, Nicola Mathieson, Tim Cooksley, Ronald Anderson, Jean Klastersky

**Affiliations:** 1 The Medical Oncology Centre of Rosebank, 129 Oxford Road, Saxonwold 2196, Johannesburg, South Africa; 2 Department of Immunology, Faculty of Health Sciences, University of Pretoria, Pretoria, South Africa; 3 Medical Oncology Department, Hospital Clinic Barcelona, Barcelona, Spain; 4 The Aga Khan University, Karachi, Pakistan; 5 Centre Hospitalier de L’Université Saint-Joseph, Beyrouth, Lebanon; 6 Institut Bordet, Bruxelles, Belgium; 7 Tumor Center Aarau and Hirslanden Clinic Aarau, Aarau, Switzerland; 8 Medical Oncology Department, University Hospital Complex of Orense, Orense, Spain; 9 Sandoz Biopharmaceuticals C/O Hexal AG, Holzkirchen, Germany; 10 The Christie NHS Foundation Trust, Manchester, UK

**Keywords:** Chemotherapy, Neutropenia, Febrile Neutropenia, Risk

## Abstract

**Purpose:**

Limited knowledge is available on the incidence of febrile neutropenia (FN) in intermediate-risk patients and the rationale for use of granulocyte colony-stimulating factor (G-CSF) in these patients. We aimed to estimate the rate at which patients associated with intermediate risk (10–20%) of FN would develop ≥ 1 episode of FN with a commonly used chemotherapy regimen in clinical practice.

**Methods:**

This prospective, real-world, observational, multinational, multicenter study (December 2016–October 2019) recruited patients with solid tumors or Hodgkin’s/non-Hodgkin’s lymphoma. Patients receiving chemotherapy with intermediate risk of FN, but not G-CSF as primary prophylaxis were included and observed for the duration of the chemotherapy (≤ 6 cycles and ≤ 30 days after the last chemotherapy administration).

**Results:**

In total, 364 patients (median age, 56 years) with 1601 cycles of chemotherapy were included in the analysis. The incidence of FN was 5% in cycle 1, 3% in cycles 2–3, and 1% in cycles 4–6. The rate of patients with ≥ 1 episode of FN was 9%, and 59% of FN events were reported during cycle 1. The rate of grade 4 neutropenia in cycle 1 was 11%, and 15% of patients experienced ≥ 1 episode of grade 4 neutropenia.

**Conclusions:**

Overall, the incidence of FN was low, with a high incidence in cycle 1 and a decrease in the subsequent cycles. These results provide the real FN risk for common chemotherapy regimens in patients generally excluded from clinical trials. Prophylactic G-CSF in intermediate-risk patients could be considered as per clinician’s judgement.

## Introduction

Despite the advances in oncology and anti-cancer therapies such as immuno-oncology and targeted therapies, chemotherapeutic agents still play a pivotal role in the management of certain solid tumors and hematological malignancies [1]. Chemotherapy-related adverse events are high and are associated with substantial morbidity and mortality [2]. One of the most frequent side effects of cytotoxic agents is neutropenia, which increases the risk of infection [1]. Febrile neutropenia (FN) is the most serious manifestation of neutropenia and a key driver of chemotherapy dose delays and/or reductions. This modification in dose intensity may impact treatment efficacy, frequently leading to hospital admissions with significant mortality due to infection-related complications [1, 3]. Furthermore, FN is accompanied by high costs attributable to the increased use of antibiotics and unplanned hospitalizations [4]. During the COVID-19 pandemic, prevention and management of severe neutropenia and FN has become even more challenging due to oncology patients’ limited or remote access to health care. Major concerns during the pandemic include delayed or interrupted standard cancer care, risk of COVID-19 infection spread in healthcare facilities and among healthcare workers, a need to keep the number of face-to-face visits to the minimum possible, and implementation of optimal FN prevention strategies [5].

The incidence of neutropenia varies between 2 and 50% and is dependent on various factors such as patient-related risk factors, type of cancer, chemotherapy, and genetic susceptibility [3, 6, 7]. FN occurs in 13–21% of patients receiving standard myelosuppressive chemotherapy regimens for metastatic solid tumors, most frequently during the first cycle (23–36%) [8].

The therapeutic action of granulocyte colony-stimulating factors (G-CSFs) results from the production and activation of neutrophils, increasing their numbers and migration in the blood. They can be used as either primary or secondary prophylaxis to reduce the risk, severity, and duration of FN, or as an adjunct to support the delivery of dose-dense (increased frequency) or dose-intense (increased dose) myelosuppressive regimens [1, 3, 7]. The use of G-CSFs as primary prophylaxis for the prevention of FN has been shown to reduce the relative risk of FN by 46% on average across 15 studies analyzed in a systematic review, with infection-related mortality and early deaths decreasing by 45% and 40%, respectively [9].

The latest updates of the leading international guidelines formulated by the American Society of Clinical Oncology (ASCO), the European Society for Medical Oncology (ESMO), the Infectious Diseases Society of America (IDSA), the National Comprehensive Cancer Network (NCCN, consensus-based guidelines), the European Organization for Research and Treatment in Cancer (EORTC), and the Multinational Association for Supportive Care in Cancer (MASCC) recommend prophylactic treatment with G-CSF in patients receiving chemotherapy associated with a high risk of FN development (≥ 20%), as well as in patients receiving chemotherapy with an intermediate risk of FN development (10–20%) in the presence of defined risk factors, including age ≥ 65 years, poor performance status, and prior FN [3, 6, 7, 10–12].

Data on FN risk are usually derived from phase III trials with inclusion criteria that limit the participation of patients with a potentially higher risk of FN. Therefore, analysis of FN incidence based on data from stringently designed prospective clinical trials encompassing patients receiving intermediate-risk chemotherapy in the real-world oncology practice is a priority.

A previous publication has described the study design and methodology of this type of prospective international multicenter observational real-world study [13]. The present report discusses the incidence of chemotherapy-induced FN in patients with an intermediate risk of FN development.

## Methods

### Study endpoints

The study's primary objective was to assess the incidence of FN (≥ 1) in patients treated with available chemotherapy regimens, which, according to published guidelines, are expected to be associated with a 10–20% risk of FN. The primary endpoint was FN incidence, defined as an absolute neutrophil count of less than 0.5 IU or 500 cells/µL of blood and temperature ≥ 38.5 °C after the first cycle of a chemotherapy regimen that is expected to be associated with intermediate risk of FN and without G-CSF prophylaxis. Secondary outcome measures included the overall incidence of FN after all chemotherapy cycles, the incidence of complicated FN after each chemotherapy cycle, and rates of morbidities (diarrhea or oral mucositis) associated with each cycle that might increase the risk of infectious complications. Other secondary endpoints included: time to the first occurrence of FN, distribution of cycle number for the first episode of FN, and impact of risk of neutropenia.

### Study design

This real-world, prospective (December 2016 to October 2019), observational, and multinational study recruited patients through a secure website. Patients were observed for the duration of the chemotherapy (≤ 6 cycles and ≤ 30 days after the last administration of chemotherapy) after they signed the informed consent forms. A total of 364 patients was enrolled from the following countries: Pakistan (*n* = 60), Lebanon (*n* = 13), Switzerland (*n* = 12), Belgium (*n* = 33), South Africa (*n* = 86), and Spain (*n* = 160).

The study initially intended to develop a risk model using the incidence of FN as a binary outcome, stratifying data by age, comorbidities, and other risk factors; however, the study was powered to have 1000 patients, but only 364 eligible patients were recruited [13].

### Patients

Adult patients diagnosed with solid tumors or Hodgkin’s/non-Hodgkin’s lymphoma, with planned administration of an accepted and available chemotherapy regimen in adjuvant, neo-adjuvant, or metastatic settings during the accrual period, were included in the study. Accepted chemotherapy regimens had an expected intermediate risk of FN in the range of 10–20% as per published guidelines [14] and study protocol [13]. Patients included in the study did not have any planned administration of G-CSF as primary prophylaxis and had not previously received another chemotherapy line. Patients who had received FN prophylaxis (with antibiotics or available G-CSF), prior high-dose chemotherapy, or stem cell transplantation were excluded. Patients scheduled to receive other chemotherapy regimens than those listed as having a FN risk of 10–20% or patients with abnormal kidney (creatinine > 1.5 × upper limit of normal) and liver function (aspartate transaminase and alanine transaminase > 2 × upper limit of normal) were also excluded.

Patients were required to provide independent, ethics committee-approved informed consent as per national laws for participation in the study. Patients were also required to comply with blood sampling. On day 8 of the first chemotherapy cycle, a blood sample for hematological counts was taken at selected sites (optional), and on day 1 of subsequent chemotherapy cycles, routine blood samples for hematological counts were taken.

### Statistical analysis

Baseline demographic variables including age, gender, and comorbidities were collected. Additional variables related to blood test parameters (neutrophils, lymphocytes, etc.) at baseline and during follow-up were also recorded/documented in a website specifically developed for the study. Comorbidities were grouped according to the Charlson comorbidity index (CCI) [15, 16] available online (https://www.mdcalc.com/charlson-comorbidity-index-cci). FN development was considered a binary outcome (ignoring the severity or risk). Descriptive statistics were used to tabulate patient characteristics. The incidence of neutropenia and FN were determined as proportions of the population.

Analyses of the data involved stratification by age (younger or older than 65 years) and examination of interactions between age, comorbidities, and other risk factors identified during the development of the logistic regression model. We then performed univariate (Chi^2^/Fisher’s exact) analyses to examine associations between patient risk factors and the prevalence of neutropenia and/or FN. Two-sided *P*-values of < 0.05 were considered statistically significant. Multivariate models included only variables that exhibited a univariate association with the dependent variable, that is, the prevalence of neutropenia (*P* < 0.05). The time to first event was estimated using the Kaplan–Meier plot and compared using the log-rank test. NCSS 2021 software for Windows (USA) was used for statistical analyses.

## Results

### Patient characteristics

A total of 364 patients was evaluable at seven centers in six countries; the median age of the patients was 56 years, and two-thirds were female (67%). Among the underlying cancers, breast cancer (53%) and colon cancer (32%) accounted for more than 80% of the cases. Notably, most of the patients received chemotherapy as part of neoadjuvant or adjuvant schemes; only about 29% of the patients had metastatic disease at baseline (Table 1).

**Table 1:** Baseline characteristics

Characteristics	*N* = 364
Age (years), median (range)	56 (17–81)
Female, n (%)	246 (67%)
Underlying cancer, n (%)	
Breast cancer	192 (52.8%)
Colorectal cancer	117 (32.1%)
Non-Hodgkin’s lymphoma	17 (4.7%)
Prostate cancer	18 (5.0%)
Germ cell tumor	9 (2.5%)
Non-small cell lung cancer	6 (1.7%)
Gastric cancer	3 (0.8%)
Esophageal cancer	2 (0.5%)
Metastatic disease	104 (28.6%)
Charlson score, n (%)	
Mild (1–2)	200 (54.9%)
Moderate (3–4)	64 (17.6%)
Severe (≥ 5)	100 (27.5%)
Weight loss ≥ 10%	
Yes	24 (6.7%)
No	311 (85.4%)
Unknown	29 (8%)

### Chemotherapy regimen modifications

The various chemotherapy regimens received by patients with a particular cancer have been previously described [13]. A total of 1601 chemotherapy cycles was administered to 364 patients. The median number of planned chemotherapy cycles was four, administered to 157 patients (43%), whereas 129 patients (36%) received six cycles of chemotherapy (Table 2). The majority of the patients (92%) completed their planned cycles of chemotherapy. Chemotherapy dose reductions and delays were observed in 78 (22%) and 148 (41%) patients, respectively. Of the total 1601 cycles, dose reductions and delays were observed in 173 (11%) and 222 (14%) cycles, respectively. The most common reasons underpinning the dose reductions and dose delays were hematological toxicity (29% and 35%), non-hematological toxicity (42% and 26%), and logistical reasons (0.02% and 27%, respectively).

**Table 2:** Administration of chemotherapy data

Number of cycles received	
Median	4 cycles
Number of patients with 4 cycles, n (%)	157 (43%)
Number of patients with 6 cycles, n (%)	129 (36%)
No chemotherapy given, n	1 patient
Reason for stopping follow-up, n (%)	
Chemotherapy complete	336 (92%)
Lost to follow-up	8 (2%)
Patient died	6 (2%)
Other reason	13 (4%)
Number of cycles with	
-Dose reduction	173 (11%)
-Delay in chemotherapy administration	222 (14%)
Number of patients with	
-Dose reduction	78 (22%)
-Delay in chemotherapy administration	148 (41%)

In total, 104 cases of diarrhea and 105 mucositis incidents were observed, of which 14% and 6%, respectively, were ≥ grade 3.

### Incidence rates of neutropenia and FN

Rates of FN were 5% in cycle 1, reduced by almost half in cycle 2 and 3 (3%, each), and 1% in cycles 4–6. The rate of patients with ≥ 1 episode of FN was 9% (95% CI: 6–12%). Site-wise incidence of FN was as follows: Aga Khan University (*n* = 22), Barcelona (*n* = 17), Bordet (*n* = 1), Rosebank (*n* = 1), and Orense (*n* = 1).

**Fig. 1:**
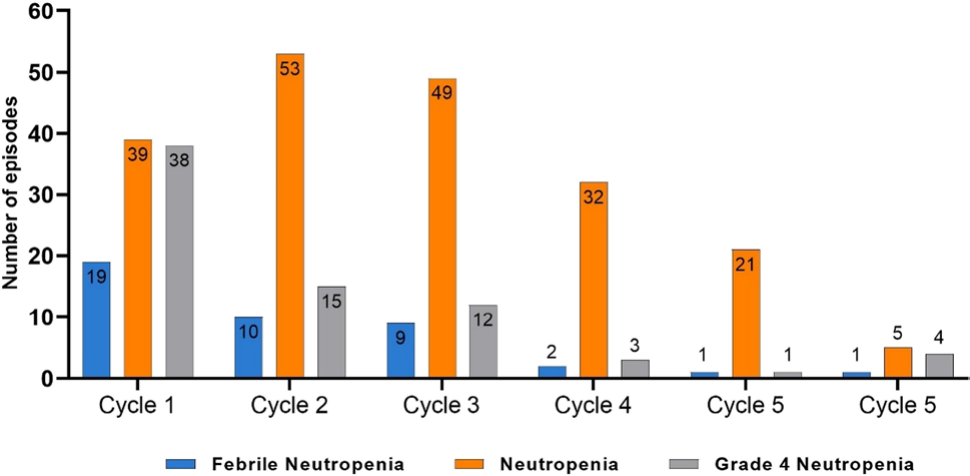
Total neutropenia and FN events per cycle

In total, 272 neutropenia episodes was observed. As shown in Table 3 and Fig. 1, the rates of all-grade neutropenia were similar: around 20% in the first three cycles (22%, 20%, and 19%, respectively); in cycles 4 and 5, the rates decreased to 12% and 15%, respectively; and a further decrease was observed in cycle 6 (7%). Except for cycle 5, an overall decrease was determined in the rates of all-grade neutropenia.

**Table 3:** Rates of neutropenia and FN

Neutropenia:	Cycle 1	Cycle 2	Cycle 3	Cycle 4	Cycle 5	Cycle 6
	*N* = 363	*N* = 342	*N* = 328	*N* = 299	*N* = 142	*N* = 129
Grade 1	11	19	24	14	8	0
Grade 2	12	19	14	10	7	4
Grade 3	16	15	11	8	6	1
Grade 4	38	15	12	3	1	4
Rate of grade 4 neutropenia, 95% CI	0.11 (0.08–0.14)	0.04 (0.02–0.07)	0.04 (0.02–0.06)	0.01 (0.002–0.03)	0.01 (< 0.01–0.04)	0.03 (< 0.01–0.08)
Rate of all grades of neutropenia, 95% CI	0.22 (0.17–0.26)	0.20 (0.16–0.25)	0.19 (0.15–0.24)	0.12 (0.09–0.16)	0.22 (0.10–0.23)	0.07 (0.03–0.13)
Febrile neutropenia:	19	10	9	2	1	1
Rate of FN, 95% CI	0.05 (0.03–0.08)	0.03 (0.01–0.05)	0.03 (0.01–0.05)	< 0.01 (< 0.01–0.02)	< 0.01 (< 0.01–0.04)	< 0.01 (< 0.01–0.04)

*CI* confidence interval, *FN* febrile neutropenia

Rates of grade 1–4 neutropenia are presented in Table 3. Patients in cycle 1 experienced a relatively higher rate of grade 4 neutropenia (11%), which decreased to 4% in cycles 2 and 3, and 1% each in cycles 4 and 5, followed by an increase in cycle 6 (3%). Overall, the rates of neutropenia of the respective grades did not show considerable consistency (Fig. 1). In 56 patients, ≥ 1 grade 4 neutropenia occurred during follow-up. The rate of patients with ≥ 1 episode of grade 4 neutropenia was 15% (95% CI: 1–20%).

Of the 32 patients who developed FN, more than half of the reported episodes (59%) occurred during cycle 1. Similar findings were observed for time to neutropenia; 48% of episodes occurred during cycle 1 among the 158 patients who developed neutropenia. Percentages of development of FN and grade 4 neutropenia by cycle are presented in Table 4.

**Table 4:** Percentages of grade 4 neutropenia and FN by cycle

	FN events (*N* = 32)	Grade 4 Neutropenia events
Cycle 1	59%	48%
Cycle 2	19%	28%
Cycle 3	16%	14%
Cycle 4	0%	6%
Cycle 5	3%	2%
Cycle 6	3%	1%

### Subgroup analysis

The overall incidence of FN was 9.5%. The age group of ≥ 65 years showed 16% incidence of grade 4 neutropenia and 8% incidence of FN. In gender‑wise distribution, the occurrence of grade 4 neutropenia and FN was similar in males (17% and 10%) and females (15% and 8%, respectively). The patients diagnosed at metastatic stage showed a greater incidence of grade 4 neutropenia (14%) and FN (7%). Other subgroup analysis outcomes are presented in Table 5.

**Table 5:** Frequency of grade 4 neutropenia and FN by patient characteristic

Patient characteristic	Grade 4 neutropenia	Febrile neutropenia
Age ≥ 65 years	14 (16%)	7 (8%)
Weight loss ≥ 10%	2 (8%)	1 (4%)
Metastatic disease	15 (14%)	7 (7%)
Charlson score – mild & moderate	42 (16%)	22 (8%)
Charlson score – severe	15 (15%)	10 (10%)
Gender – male	20 (17%)	12 (10%)
Gender – female	37 (15%)	20 (8%)
Creatinine (mg/dL)	0.5	0.79

## Discussion

Although current chemotherapy regimens for solid tumors are well tolerated in most tumor types, they are sometimes associated with severe and life-threatening events such as FN [4]. To our knowledge, our study is the first prospective, real-world, observational multinational study that evaluated the incidence of FN in different cycles of chemotherapy in patients with intermediate-risk FN and analyzed the associated dose reduction and treatment delays in chemotherapy in different types of cancers. The study excluded patients receiving G-CSF as primary prophylaxis.

Investigator-reported incidence of FN varies considerably for the same chemotherapy regimen. The results of this study demonstrated different outcomes from different sites/countries, with The Aga Khan University and Barcelona representing 58% of the cohort and reporting 93% of the FN incidents, whereas Rosebank and Bordet representing 33% of the cohort and reporting only 5% of the FN incidents. This difference could be attributed to patient selection, different patient characteristics, different chemotherapy regimens, or differential use of G-CSF for prophylaxis after the second chemotherapy cycle.

Evidence suggests that FN rates in the real-world setting may be higher than those reported in randomized controlled trials (RCTs), a possible reason being the stringent eligibility criteria for entry into RCTs. In a systematic review and meta-analysis of 65 observational (*n* = 7812 patients) and 110 RCT (*n* = 42,257 patients) cohorts involving 29 breast cancer chemotherapy regimens, the unadjusted FN rate was 11.7% and 7.9% in the observational and RCT cohorts, respectively [17]. The present study reflected this number, with an FN rate of 11.6%. Our data suggested that FN incidence is similar to the FN rates of real-world data and other RCTs; however, our study might have been biased due to the exclusion of patients receiving G-CSF as primary prophylaxis.

This is a real-world study, as opposed to G-CSF Phase III clinical trials, in which clinical and laboratory parameters are recorded frequently. In real-world studies, these parameters are recorded as frequently. The main aim of this research was to establish real-world incidence encompassing the world's various regions. The study design and the frequency of the investigations were done to resemble clinical practice as closely as possible in many settings worldwide. Frequent testing might indeed overestimate the incidence of FN, resulting in overtreatment. Additionally, this approach might decrease quality of life and increase costs.

The rate of patients with ≥ 1 episode of FN was 9% in the present study. This rate was higher than results observed in a previously published RCT [18] and a real-world study [19]. In the RCT, the overall proportion of patients having ≥ 1 occurrence of FN was 5.8% for the 7/10-day group [18]. Similar results were observed in the real-world study in which the rate of patients who developed ≥ 1 episode of FN was 6.1% [19]. The higher number of chemotherapy cycles administered in the present study (*n* = 1601), compared to the RCT (*n* = 1302) and the real-word study (*n* = 912), might be a possible reason for this difference. Other reasons could be related to patient heterogeneity, type of regimen used, different inclusion criteria, different tumor types, or lack of control for dose reductions and dose delays.

In the present analysis, the rate of episodes of FN in each cycle varied; 5% were encountered in cycle 1, followed by 3% and < 1% in cycles 2 and 6, respectively. A similar pattern, but a higher rate of FN, was noted in an observational study; of 19 patients, the first (36.36%) and second (22.72%) cycles of chemotherapy exhibited a higher incidence of FN cases than other chemotherapy cycles [20]. Culakova et al. reported 9.7% incidence of FN in cycle 1, followed by 5.7% and 3.8% in cycles 2 and 3, respectively [21]. A decrease in neutropenic events was observed in subsequent cycles because of clinical interventions such as reductions in chemotherapy dose intensity and/or additional use of supportive care measures (G-CSF prophylaxis or antibiotics) [21]. Reduced relative dose intensity was more common for older patients, those with poor performance status, and obese patients [21]. The present study results correlate with those of previous studies in that the incidence was higher in cycles 1 and 2, but varied in the cycles that followed [22].

Currently, chemotherapy-induced myelosuppression, which reduces chemotherapy dose intensity and potentially limits therapeutic efficacy, is managed with chemotherapy dose delays/reductions and lineage-specific supportive care interventions [23]. The present analysis observed dose reductions and delays in 78 (22%) and 148 (41%) patients, respectively. These results were in line with previously published data. Results from one RCT [18] showed that the overall proportion of patients having ≥ 1 occurrence of either chemotherapy dose delay or reduction or discontinuation was 37.64% and 39.42% for the 5- and 7/10-day groups, respectively [18]. In a retrospective cohort study, considerable variability in dose delay of ≥ 7 days was seen among cancer types and among chemotherapy regimens, which varied from 9.3% to 16.3% for different cancer types [24].

In this study, a high incidence of treatment dose reductions and dose delays, including patients receiving adjuvant treatment, were recorded. It should be noted that this observational study did not have specific recommendations regarding dose modifications; therefore, these reductions and delays reflect clinicians' and patients' preferences.

In the setting of the COVID-19 pandemic, where clinicians aim to minimize patients’ risk of potential infection and need for hospital visits, the prophylaxis and management of FN is of particular importance. As FN can result in the need for hospitalization, ESMO recommends that physicians consider using regimens with a lower risk of FN in patients who are not being treated with curative intent [25]. ESMO [25] and NCCN [26] also suggest expanding the indication for G-CSF after chemotherapy to lower the risk of FN but acknowledge that this may require additional outpatient visits. Similar to the NCCN and ESMO recommendations, the American Society of Clinical Oncology (ASCO) [27] recognizes that during the COVID-19 pandemic, it may be reasonable for patients at lower risk of FN to be prescribed growth factors [27]. Furthermore, with wider availability of biosimilars for G-CSF, the cost-effectiveness of prophylactic treatment in intermediate-risk patients may be a rationale for consideration.

In the current study, we were unable to develop a risk model for FN in this set of chemotherapy regimens with an expected FN rate of 10–19% or identify a particular set of patients at higher risk of FN owing to slow recruitment and low patient numbers. The primary aim of the current study was to develop a model for predicting FN in oncology patients receiving chemotherapy with intermediate risk (10–20% risk) involving 1000 patients in 20 centers worldwide. This recruitment was not achieved; however, we report a secondary endpoint of the study describing the incidence of FN in 364 patients recruited.

The heterogeneity of the patient population in terms of tumor type and the wide range of chemotherapy agents made the interpretation of risk factors difficult. Further research on patient populations with similar tumor types and chemotherapy schedules may help stratify the patients by age and comorbidities. With wider use of mixed anticancer regimens combining chemoimmunotherapy with targeted therapies for many cancer types, the incidence of FN may be further reduced. Prophylactic G-CSF in intermediate-risk patients may be considered according to the clinician’s judgement. A quality-of-life component was also recorded and will be reported separately.

The study limitations are mainly the lack of data about anticancer treatments including concomitant chemo-immuno therapies (current regimens for non-small cell lung cancer and other cancers) and COVID-19 infection because of the study period. Other limitations include lack of data related to financial status or healthcare system, G-CSF coverage, and unavailability of data about patient preferences and specific education of patients regarding FN at each participating center.

In conclusion, in patients receiving chemotherapy regimens with an intermediate risk of FN, the rate of FN was on par with other RCTs and real-world studies. The rates were high in cycles 1 and 2 but varied in the subsequent cycles. This large observational, real-world study found that cancer patients with intermediate-risk of FN (10–20%) can be treated prophylactically with G-CSF in alignment with the current guidelines. Overall, this prospective study provides insights and real-world evidence to support the implementation of the current guidelines in daily practice.

## Data Availability

Novartis supports the publication of scientifically rigorous analysis that is relevant to patient care, regardless of a positive or negative outcome. Qualified external researchers can request access to anonymized patient-level data, respecting patient-informed consent, through www.clinicalstudydatarequest.com, according to requirements noted on the web portal.

## References

[1] Tralongo AC, Antonuzzo A, Pronzato P, Sbrana A, Turrini M, Zoratto F, Danova M (2020). Management of chemotherapy-induced neutropenia in patients with cancer: 2019 guidelines of the Italian Medical Oncology Association (AIOM). Tumori Journal.

[2] Kuderer NM, Desai A, Lustberg MB, Lyman GH (2022) Mitigating acute chemotherapy-associated adverse events in patients with cancer. Nat Rev Clin Oncol 19(11):681-697. 10.1038/s41571-022-00685-3 10.1038/s41571-022-00685-3 36221000

[3] Klastersky J, De Naurois J, Rolston K, Rapoport B, Maschmeyer G, Aapro M, Herrstedt J (2016). Management of febrile neutropaenia: ESMO clinical practice guidelines. Ann Oncol.

[4] Kasi PM, Grothey A (2018). Chemotherapy-induced neutropenia as a prognostic and predictive marker of outcomes in solid-tumor patients. Drugs.

[5] Cooksley T, Font C, Scotte F, Escalante C, Johnson L, Anderson R, Rapoport B (2021). Emerging challenges in the evaluation of fever in cancer patients at risk of febrile neutropenia in the era of COVID-19: a MASCC position paper. Support Care Cancer.

[6] BL R Febrile Neutropenia - Guideline Update and Approach to Management of Both High and Intermediate-Risk Patients. https://mascc.org/wp-content/uploads/2022/04/1114_Rapoport_Strauss-1-2_Sat.pdf. Accessed November 23 2022

[7] NCCN Clinical Practice Guidelines in Oncology (2022) Prevention and Treatment of Cancer-Related Infections, version 1. 2022. National Cancer Comprehensive Network website. https://www.nccn.org/professionals/physician_gls/pdf/infections.pdf. Accessed November 23 2022 10.6004/jnccn.2016.0093 27407129

[8] Weycker D, Li X, Edelsberg J, Barron R, Kartashov A, Xu H, Lyman GH (2015). Risk and consequences of chemotherapy-induced febrile neutropenia in patients with metastatic solid tumors. J Oncol Pract.

[9] Kuderer NM, Dale DC, Crawford J, Lyman GH (2007) Impact of primary prophylaxis with granulocyte colony-stimulating factor on febrile neutropenia and mortality in adult cancer patients receiving chemotherapy: a systematic review. J Clin Oncol 25(21):3158–67. 10.1200/JCO.2006.08.8823 10.1200/JCO.2006.08.8823 17634496

[10] Aapro M, Bohlius J, Cameron DA, Dal Lago L, Donnelly JP, Kearney N, Lyman G, Pettengell R, Tjan-Heijnen V, Walewski J (2011). 2010 update of EORTC guidelines for the use of granulocyte-colony stimulating factor to reduce the incidence of chemotherapy-induced febrile neutropenia in adult patients with lymphoproliferative disorders and solid tumours. Eur J Cancer.

[11] Freifeld AG, Bow EJ, Sepkowitz KA, Boeckh MJ, Ito JI, Mullen CA, Raad II, Rolston KV, Young J-AH, Wingard JR (2011). Clinical practice guideline for the use of antimicrobial agents in neutropenic patients with cancer: 2010 update by the Infectious Diseases Society of America. Clin Infect Dis.

[12] Smith TJ, Bohlke K, Lyman GH, Carson KR, Crawford J, Cross SJ, Goldberg JM, Khatcheressian JL, Leighl NB, Perkins CL (2015). Recommendations for the use of WBC growth factors: American Society of Clinical Oncology clinical practice guideline update. J Clin Oncol.

[13] Rapoport BL, Aapro M, Paesmans M, Van Eeden R, Smit T, Krendyukov A, Klastersky J (2018) Febrile neutropenia (FN) occurrence outside of clinical trials: occurrence and predictive factors in adult patients treated with chemotherapy and an expected moderate FN risk. Rationale and design of a real-world prospective, observational, multinational study. BMC Cancer 18 (1):1–8 10.1186/s12885-018-4838-z PMC6154917 30249215

[14] Li Y, Family L, Yang S-J, Klippel Z, Page JH, Chao C (2017). Risk of febrile neutropenia associated with select myelosuppressive chemotherapy regimens in a large community-based oncology practice. J Natl Compr Canc Netw.

[15] Charlson ME, Pompei P, Ales KL, MacKenzie CR (1987). A new method of classifying prognostic comorbidity in longitudinal studies: development and validation. J Chronic Dis.

[16] Quan H, Li B, Couris CM, Fushimi K, Graham P, Hider P, Januel J-M, Sundararajan V (2011). Updating and validating the Charlson comorbidity index and score for risk adjustment in hospital discharge abstracts using data from 6 countries. Am J Epidemiol.

[17] Truong J, Lee EK, Trudeau ME, Chan KK (2016). Interpreting febrile neutropenia rates from randomized, controlled trials for consideration of primary prophylaxis in the real world: a systematic review and meta-analysis. Ann Oncol.

[18] Clemons M, Fergusson D, Simos D, Mates M, Robinson A, Califaretti N, Zibdawi L, Bahl M, Raphael J, Ibrahim MFK, Fernandes R, Pitre L, Aseyev O, Stober C, Vandermeer L, Saunders D, Hutton B, Mallick R, Pond GR, Awan A, Hilton J (2020). A multicentre, randomised trial comparing schedules of G-CSF (filgrastim) administration for primary prophylaxis of chemotherapy-induced febrile neutropenia in early stage breast cancer. Ann Oncol.

[19] Palukuri NR, Yedla RP, Bala SC, Kuruva SP, Chennamaneni R, Konatam ML, Gundeti S (2020). Incidence of febrile neutropenia with commonly used chemotherapy regimen in localized breast cancer. South Asian J Cancer.

[20] Philip MLSN, Sebastian MA, Mateti UV (2019). Assessment of chemotherapy-induced febrile neutropenia in cancer patients. IJMPO.

[21] Culakova E, Poniewierski MS, Wolff DA, Dale DC, Crawford J, Lyman GH (2015). The impact of chemotherapy dose intensity and supportive care on the risk of febrile neutropenia in patients with early stage breast cancer: a prospective cohort study. Springerplus.

[22] Hosmer W, Malin J, Wong M (2011). Development and validation of a prediction model for the risk of developing febrile neutropenia in the first cycle of chemotherapy among elderly patients with breast, lung, colorectal, and prostate cancer. Support Care Cancer.

[23] Lyman GH, Kuderer NM, Aapro M (2021). Improving outcomes of chemotherapy: established and novel options for myeloprotection in the COVID-19 era. Front Oncol.

[24] Kawatkar AA, Farias AJ, Chao C, Chen W, Barron R, Vogl FD, Chandler DB (2017). Hospitalizations, outcomes, and management costs of febrile neutropenia in patients from a managed care population. Support Care Cancer.

[25] European Society for Medical Oncology (2020) Supportive Care Strategies During the COVID-19 Pandemic. https://www.esmo.org/guidelines/cancer-patient-management-during-the-covid-19-pandemic/supportivecare-in-the-covid-19-era. Accessed November 23 2022

[26] Griffiths EA, Alwan LM, Bachiashvili K, Brown A, Cool R, Curtin P, Geyer MB, Gojo I, Kallam A, Kidwai WZ, Kloth DD, Kraut EH, Lyman GH, Mukherjee S, Perez LE, Rosovsky RP, Roy V, Rugo HS, Vasu S, Wadleigh M, Westervelt P, Becker PS (2020) Considerations for use of hematopoietic growth factors in patients with cancer related to the COVID-19 pandemic. J Natl Compr Canc Netw 1–4. 10.6004/jnccn.2020.7610 10.6004/jnccn.2020.7610 PMC9730290 32871558

[27] American Society of Clinical Oncology (2020) COVID-19 Patient Care Information. Cancer Treatment & Supportive Care. https://www.asco.org/asco-coronavirus-resources/care-individuals-cancer-during-covid-19/cancer-treatment-supportive-care. Accessed November 23 2022

